# Pathophysiological role of BACH transcription factors in digestive system diseases

**DOI:** 10.3389/fphys.2023.1121353

**Published:** 2023-05-09

**Authors:** Qianben Song, Xin Mao, Mengjia Jing, Yu Fu, Wei Yan

**Affiliations:** ^1^ Department of Gastroenterology, Tongji Hospital of Tongji Medical College, Huazhong University of Science and Technology, Wuhan, Hubei, China; ^2^ Institute of Liver and Gastrointestinal Diseases, Tongji Hospital of Tongji Medical College, Huazhong University of Science and Technology, Wuhan, Hubei, China; ^3^ Department of Gastroenterology, Union Hospital, Tongji Medical College, Huazhong University of Science and Technology, Wuhan, Hubei, China

**Keywords:** BACH1, BACH2, cancer, digestive system diseases, transcription factor

## Abstract

BTB and CNC homologous (BACH) proteins, including BACH1 and BACH2, are transcription factors that are widely expressed in human tissues. BACH proteins form heterodimers with small musculoaponeurotic fibrosarcoma (MAF) proteins to suppress the transcription of target genes. Furthermore, BACH1 promotes the transcription of target genes. BACH proteins regulate physiological processes, such as the differentiation of B cells and T cells, mitochondrial function, and heme homeostasis as well as pathogenesis related to inflammation, oxidative-stress damage caused by drugs, toxicants, or infections; autoimmunity disorders; and cancer angiogenesis, epithelial-mesenchymal transition, chemotherapy resistance, progression, and metabolism. In this review, we discuss the function of BACH proteins in the digestive system, including the liver, gallbladder, esophagus, stomach, small and large intestines, and pancreas. BACH proteins directly target genes or indirectly regulate downstream molecules to promote or inhibit biological phenomena such as inflammation, tumor angiogenesis, and epithelial-mesenchymal transition. BACH proteins are also regulated by proteins, miRNAs, LncRNAs, labile iron, and positive and negative feedback. Additionally, we summarize a list of regulators targeting these proteins. Our review provides a reference for future studies on targeted drugs in digestive diseases.

## Introduction

**FIGURE 1:**
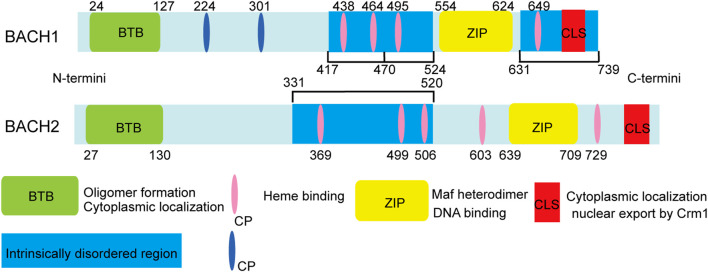
Structure and function of mouse BACH1 and BACH2. The amino acid positions of cysteines are indicated by numbers. The intrinsically disordered heme binding region of BACH1 can be divided into three functional parts: the first part (417–470), including two CP motifs, affects activator recruitment by binding to the local conformation of the five-coordinate heme and is involved in other protein-protein interactions; the second part (471–524), including one CP motif, participates in dissociation from DNA; and the last part (331–520), which contains one CP motif, may participate in destabilization of the BACH1/MAFK heterodimer and subsequent DNA dissociation.

Bric-a-brac-Tramtrack-Broad (BTB) and cap “n” collar (CNC) homologue (BACH) transcription factors, including BACH1 and BACH2, are members of the basic leucine zipper (bZIP) family (Oyake et al., 1996). BACH transcription factors comprise a bZIP domain, BTB domain, cysteine–proline (CP) motifs, cytoplasmic localization signal (CLS), and intrinsically disordered regions (IDRs) (Igarashi et al., 2017). The structure of the BACH proteins and the functions corresponding to each structure are shown in Figure 1.

**FIGURE 2:**
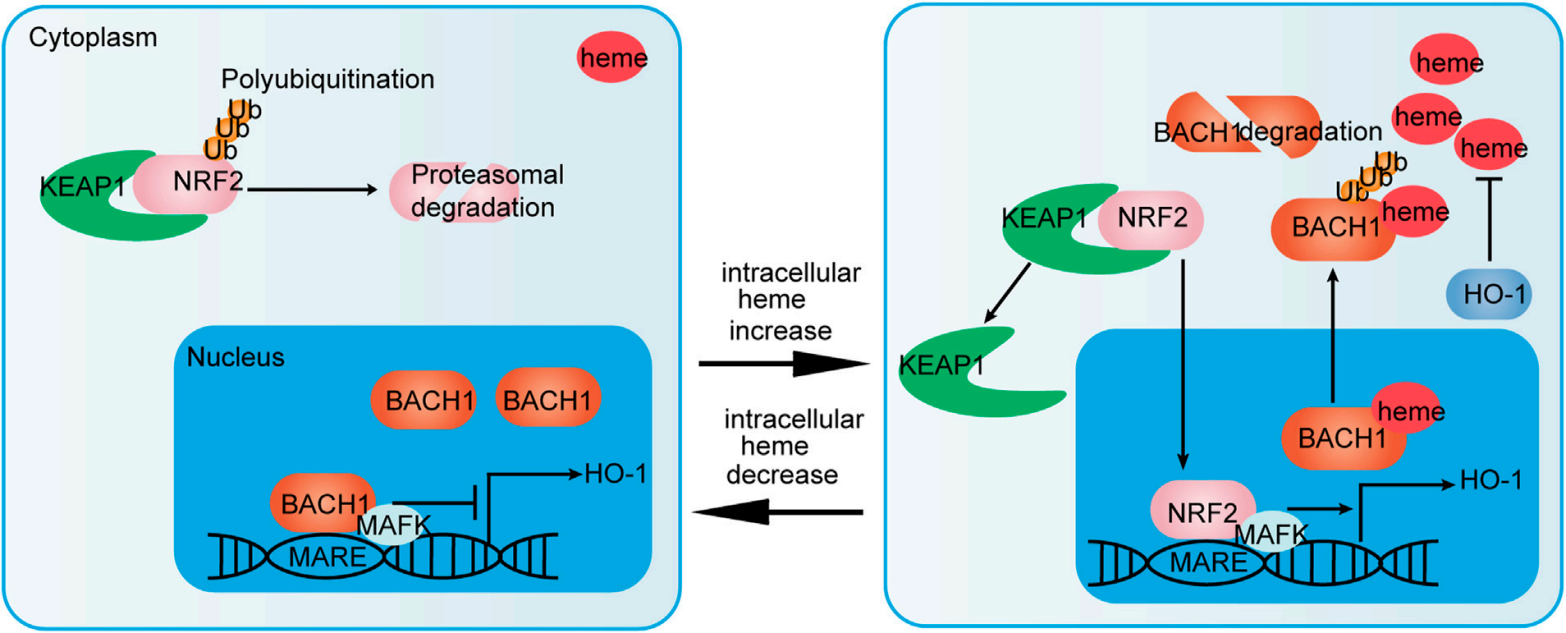
NRF2/BACH1 pathway that regulates HO-1 expression. NRF2/MAFK heterodimers activate HO-1 expression. Activation of HO-1 expression is inhibited by BACH1/MAFK heterodimers at low heme concentrations. Meanwhile, NRF2 is polyubiquitinated and degraded by interacting with Kelch-like ECH-associated protein 1 (KEAP1) (left). At high heme concentrations, heme binds to BACH1. Then, BACH1 dissociates from MAF recognition elements (MARF), is exported to the cytoplasm and ubiquitinated for degradation. NRF2 nuclear accumulation via heme-mediated disruption of NRF2/KEAP1 complexes promotes the formation of NRF2/MAFK heterodimers (right). In this way, the NRF2/BACH1 pathway affects the expression of HO-1 and regulates heme homeostasis.

The bZIP domain of BACH transcription factors mediates heterodimer formation with small musculoaponeurotic fibrosarcoma (MAF) proteins MAFF, MAFG, and MAFK, and controls DNA binding to repress the transcription of the target gene (Zhou et al., 2016). BACH transcription factors can identify and bind to MAF recognition elements, which are well conserved in both mouse and human gene promoters (Igarashi and Watanabe-Matsui, 2014). Consequently, BACH1 can block the transcription of several oxidative stress-response genes, including heme oxygenase-1 (HO-1/HMOX1), and NADPH and quinone oxidoreductase 1 (NQO1). Accumulated nuclear factor erythroid 2-Like 2 (NRF2) competes with BACH1 to form a heterodimer with MAF proteins. In this way, NRF2 activates the transcription of genes, such as *H O -1* which encodes an anti-oxidant defense enzyme. HO-1 degrades heme into ferrous iron, biliverdin, and carbon monoxide (Zhang X. et al., 2018). Figure 2 illustrates the regulation of HO-1 via the BACH1/NRF2 pathway. In addition to repressing HO-1 transcription, BACH2 and bZIP ATF-like transcription factor form heterodimer that binds to the activator protein 1 (AP-1) motif, which contains regulatory regions of Th2 cytokine gene loci (Kuwahara et al., 2016).

The BTB domain, also known as the pox virus and zinc finger domain, is located at the N-terminus of BACH proteins (Oyake et al., 1996). The analysis of BTB proteins from 17 eukaryotic species revealed that the structure of BTB is highly conserved (Stogios et al., 2005). The BTB domain has a unique three-dimensional fold with a large protein–protein interaction surface and highly variable exposed residues, thereby allowing dimerization and oligomerization, as well as interaction with several other proteins (Perez-Torrado et al., 2006). BACH1 inhibits angiogenesis and the enhancer activity of locus control region in a BTB domain-dependent manner. Moreover, the BTB domain of BACH1 is necessary for the formation of large-looped DNA between two distant binding sites *in vitro*. The formation of BACH1/MAFK heterodimers through the BTB domain generates a multivalent DNA binding complex (Yoshida et al., 1999; Jiang L. et al., 2020). The BTB domain of BACH2 coordinates the interaction with HDAC3-containing co-repressor complexes to regulate the protein levels of Blimp-1 via epigenetic modifications in B cells (Tanaka et al., 2016).

The activity of the CLS, which is a highly conserved structure at the C-terminus of the BACH proteins, depends on a segment of nonhydrophobic amino acids. Oxidative stress disrupts the activity of CLS to induce the nuclear accumulation of BACH2 (Hoshino et al., 2000). Cadmium induces HO-1 expression and promotes the CLS-mediated nuclear export of BACH1 and BACH2 (Suzuki et al., 2003). Furthermore, the nuclear export of BACH1 depends on the activities of extracellular signal-regulated kinase-1/2 (ERK1/2) and exporter chromosome maintenance 1 (Crm1/Exportin-1) (Suzuki et al., 2003). Xpo1, also known as Crm1, binds to BACH1 and exports it to the cytoplasm, which promotes the formation of transcriptional complexes with NRF2 and small MAF proteins (Suzuki et al., 2004).

BACH1 consists of six CP motifs, whereas BACH2 consists of five CP motifs. BACH1 and BACH2 directly bind to heme through the C-terminal region with four and five CP motifs, respectively (Ogawa et al., 2001; Watanabe-Matsui et al., 2011). BACH proteins consist of two modes of heme binding: five- and six-coordination modes (Hira et al., 2007; Watanabe-Matsui et al., 2011). The five-coordinate mode is unique to BACH1 CP motifs, whereas six-coordination mode binding occurs nonspecifically (Segawa et al., 2019b). Heme binds multiple CP motifs within the IDRs of the BACH proteins, thereby inhibiting their DNA-binding activity and inducing nuclear export, polyubiquitination, and degradation of the BACH proteins (Zhou et al., 2016; Segawa et al., 2022). BACH1 degradation is induced by the heme binding of E3 ubiquitin ligase Hoil-1, Fbxl17 and Fbxo22. However, the specific mechanisms through which heme induces BACH2 polyubiquitination and degradation remain unknown (Zenke-Kawasaki et al., 2007; Tan et al., 2013; Lignitto et al., 2019).

IDRs generally lack long hydrophobic amino acid sequences, resulting in their inability to form a well-organized hydrophobic core comprising a structural domain, but they still perform certain biological activities (Oldfield and Dunker, 2014). Furthermore, IDRs not only recognize and recruit partners, such as proteins, but also participate in conformational changes, and post-translational modifications (van der Lee et al., 2014). Heme binds to BACH1 C-terminal IDRs through a conformational change (Segawa et al., 2019a). A recent study showed that different CP motifs in the heme-bound BACH1 IDRs play roles in co-activator recruitment, dissociation from DNA, and heterodimer destabilization (Segawa et al., 2022). In intrinsically-disordered heme binding regions of BACH2, heme binding induces disordered conformational alterations and more compact BACH2 IDRs conformations (Watanabe-Matsui et al., 2015; Suenaga et al., 2016).

**FIGURE 3:**
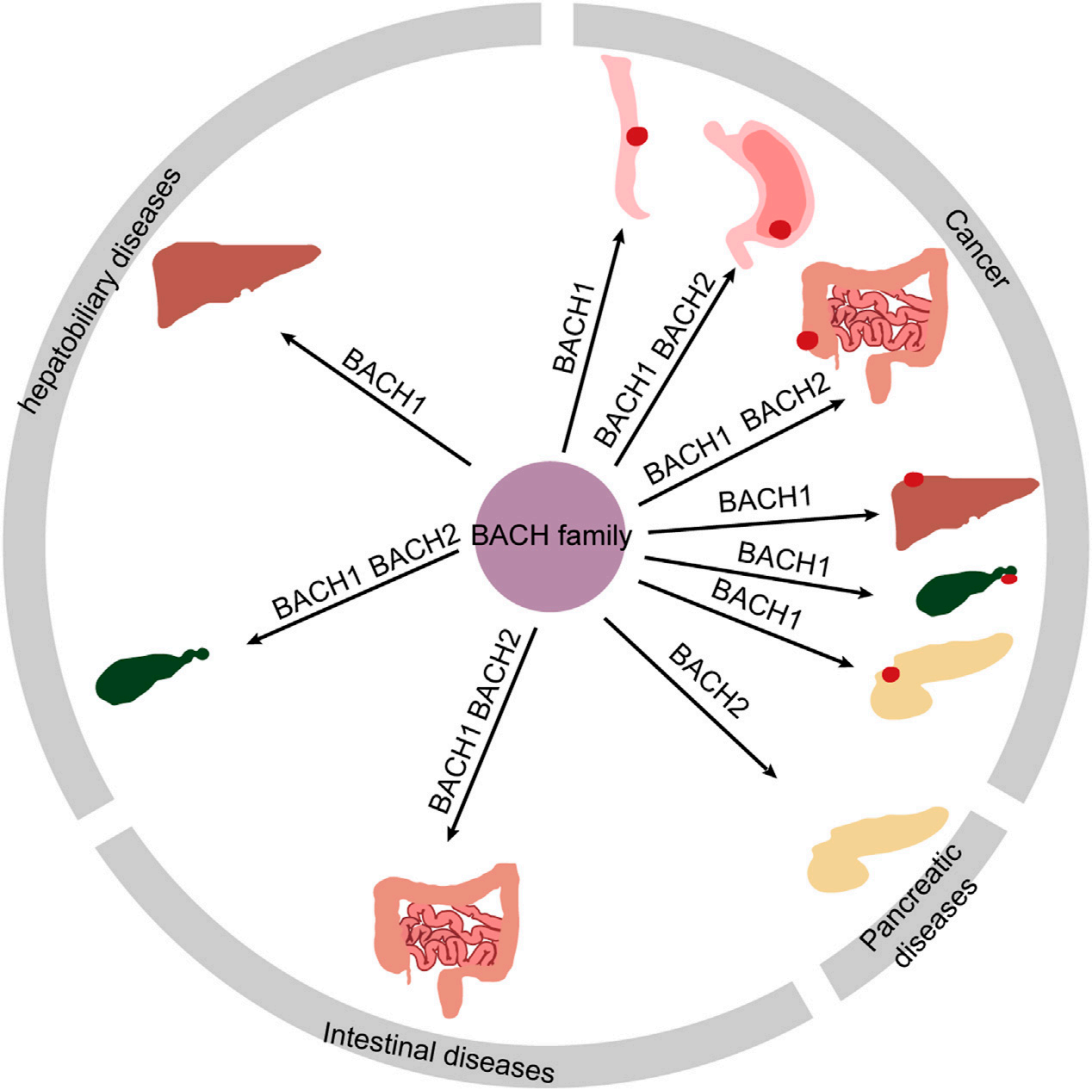
Overview diagram showing digestive disorders associated with BACH proteins.

BACH proteins were identified in 1996, and since then a growing number of studies have shown that BACH transcription factors are widely expressed in human tissues. These transcription factors regulate the development and function of innate and adaptive immune systems; progression, and metabolism of many cancers; infection; apoptosis; angiogenesis; lymphangiogenesis; oxidative stress injury; and senescence, and pluripotency of embryonic stem cells (Igarashi et al., 2017; Zhang X. et al., 2018; Cohen et al., 2020; Niu et al., 2021; Padilla and Lee, 2021; Chu et al., 2022). For instance, BACH2 controls CD4^+^ and stem-like CD8^+^ T-cell differentiation (Watanabe-Matsui et al., 2011; Yao et al., 2021). Both BACH1 and BACH2 inhibit the myeloid program to promote B cell development (Itoh-Nakadai et al., 2014). In this review, we summarize the pathophysiological role of BACH proteins in digestive system diseases including hepatitis C virus (HCV) infection, nonalcoholic steatohepatitis (NASH), hepatic and intestinal injury induced by drugs and other causes, immune-mediated intestinal diseases [inflammatory bowel disease (IBD) and celiac diseas], biliopancreatic diseases, and digestive system cancers (Table 1). The relationship between BACH proteins and the above diseases is shown in Figure 3. We find that BACH1 aggravates benign diseases mainly by inhibiting the expression of downstream molecules, such as transcriptional inhibition of HO-1, and promotes cancer progression of the digestive system mainly by upregulating the expression of downstream molecules, such as transcriptional activation of IGF1R. In addition, BACH1 has different regulatory effects in different cells or different microenvironments of the same cells. BACH2 affects tumor progression and immune-related benign diseases by regulating the differentiation and function of immune cells.

**TABLE 1:** Level and effect of BACH in digestive system diseases.

Name	Organs	Disease	Alteration	Function	References
BACH1	Esophagus	ESCC	Upregulated	Promotes the proliferation, invasion and metastasis by inducing EMT and angiogenesis	Jiang et al. (2015); Xie et al. (2023)
				Inhibits biosynthesis of MUFAs by transcriptionally inhibiting *SCD1* gene to induce ferroptosis, thereby promoting lymphatic metastasis and inhibiting hematogenous metastasis	
BACH1	stomach	GC	Upregulated	Promotes macrophage-dependent GC progression via polarization into M2 macrophages	Fang and Lu. (2020); Yang et al. (2022)
BACH2	Stomach	GC	Upregulated	Associated with poor prognosis in patients with GC of MSI-H	Tian et al. (2020); Haam et al. (2014)
				BACH2 deficiency promotes GC cell proliferation *in vitro*	
BACH1	Bowel	CRC	Dynamically altered	Enhances CRC cell migration, invasion by increasing the expression of STARD8, TIAM2, MMP-1, MMP-9, MMP-13, SNAIL1, CXCR4, and HMGA2	Chang et al. (2020); El-Deek et al. (2019); Zhu et al. (2018); Chen et al. (2022)
				Promotes or have no effect on proliferation	
BACH1	Bowel	IBD		BACH1-deficiency macrophages inhibit TNBS-induced colitis by promoting M2 phenotype	Harusato et al. (2013); Takagi et al. (2018); Pradhan et al. (2022)
				BACH1 deficiency promotes NLRP3 inflammasome activation and affects mitochondrial function	
				Reduced BACH1 expression protects the intestinal mucosa in DSS-induced colitis mouse model	
		Indometha-cin induced intestinal injury		Attenuates injury via suppressing inflammation and apoptosis	Harusato et al. (2011)
BACH2	Bowel	CRC		Promotes immune homeostasis, durable tumor immunosuppression and metastasis by regulating Treg and NK cells	Grant et al. (2020); Li et al. (2022)
		UC(IBD)	Upregulated	Significantly associated with UC	Ding et al. (2021)
		CD (IBD)		CD susceptibility gene	Romani et al. (2021); Laffin et al. (2018)
				Closely related to the postoperative recurrence in patients with CD	
		IBD		Regulates CD161+ Treg cells to enhance wound healing of epithelium	Povoleri et al. (2018)
		CeD	Downregulated	Associated with CeD pathogenesis	Medrano et al. (2019); Quinn et al. (2015)
BACH1	Liver	HCC	Upregulated	Promotes growth, metastasis, or cellular inflammation through transcriptional activation of IGF1R, PKT and HK2 or influencing P53 pathway	Xie et al. (2022); Sun et al. (2021); Du et al. (2022); Sun et al. (2020a); Xu et al. (2016); Zhao et al. (2022)
				Suppresses autophagy, proliferation, metastasis by influencing P53 pathway or targeting *TKT* gene	
				Regulates glucose and glutathione metabolism	
BACH1	Liver	HCV		Deficient BACH1 inhibits HCV replication	Ghaziani et al. (2006); Chen et al. (2019)
				Influences cytotoxic effects of HCV proteins	
		NASH		Inhibition of BACH1 reduces steatohepatitis and fibrosis	Inoue et al. (2011)
		ATDH		SNPs importantly associated with ATDH	Zhang et al. (2018a)
BACH1	Liver	APAP-induced liver injury	Upregulated	Involved in oxidative stress promoting liver damage	Abo El-Magd and Eraky (2020)
		Sepsis-induced liver injury	Dynamically altered	Knockdown of BACH1 attenuates liver injury via increasing hepatic blood flow and decreasing oxidative stress and inflammation	Tanioka et al. (2021); Cai et al. (2022)
BACH1	Liver	Ethinylestr-adiol induced cholestasis		Influences bile flow and urinary bile acid clearance	Muchova et al. (2015)
	Bile duct				
BACH1	Bile duct	CCA	Downregulated	Inhibits the transcription of genes encoding proteasome subunits	Jiang et al. (2020b); Liu et al. (2022b)
BACH2	Bile duct	PSC		Associated with the susceptibility	Liu et al. (2013b)
BACH1	Pancreas	Pancreatic cancer	Upregulated or Downregulated	Promotes EMT by increasing iron and the expression of vimentin and SNAI2 and decreasing E-cadherin, FOXA1, CLDN3, and CLDN4 expression	Kim et al. (2021); Sato et al. (2020); Liu et al. (2022); Huang et al. (2018); Liu et al. (2022)
				Inhibits EMT by increasing E-cadherin and ZO-1 expression and decreasing ZEB1, vimentin and Slug expression	
				Suppresses cell growth and angiogenesis	
				SNPs is related with worse overall survival and resistant to gemcitabine	
BACH2		Chronic pancreatitis	Downregulated	BACH2 inhibition promotes polarization toward Th17 cells and a higher inflammatory response	Sasikala et al. (2018)
				Associated with advanced clinical features	

Abbreviations: ESCC, Esophageal squamous cell carcinoma; GC, Gastric cancer; CRC, Colorectal cancer; HCC, Hepatocellular carcinoma; CCA, Cholangiocarcinoma; NASH, Nonalcoholic steatohepatitis ; ATDH, Anti-tuberculosis drug-induced hepatotoxicity; APAP, Acetaminophen; UC, Ulcerative colitis; CD, Crohn’s disease; IBDs, Inflammatory bowel diseases; CeD, Coeliac disease; PSC, Primary sclerosing cholangiti; MSI-H, microsatellite instability-high status microsatellite instability-high status; MUFAs, monounsaturated fatty acids.

## BACH proteins and benign diseases of the digestive system

### Hepatobiliary diseases

#### Hepatitis C virus

The incidence of HCV has been decreasing; however, there was still approximately 56.8 million HCV infections worldwide in 2020 (Polaris Observatory HCV Collaborators, 2022). One study found that reduced BACH1 and elevated HO-1 levels played critical roles in reducing the cytotoxicities of HCV proteins in hepatocellular carcinoma (HCC) cells (Ghaziani et al., 2006). BACH1 is involved in the mechanism of action of miR-let-7c, miR-196, and miR-122 inhibiting HCV replication *in vitro*. Both miR-let-7c and miR-196 suppress BACH1 expression by directly acting on the 3′-UTR of BACH1 mRNA to downregulate the levels of HCV protein. A decline in BACH1 expression reduces HCV replication by promoting HO-1 expression to inhibit the activity of viral protease and stimulate an antiviral interferon response. Whether miR-122 suppresses HCV replication through the above mechanism remains to be investigated (Shan et al., 2007; Hou et al., 2010; Chen et al., 2019). Decreased HO-1 expression in patients with HIV/HCV co-infection may indicate a worse prognosis. However, HO-1 expression is positively correlated with BACH1 and miR-122 expression in patients with HCV (Jabłonowska et al., 2014). The expression of hepatic NRF2 and BACH1, and serum HO-1 result in the destructive increase in acetaminophen (APAP)-induced liver injury (Abo El-Magd and Eraky, 2020). HO-1 expression in patients with HCV may be affected by NRF2, which competes with BACH1 for binding to the HO-1 gene. Further, Legalon-SIL (LS) and statins decreased HCV replication and influenced the NRF2/BACH1/HO-1 pathway (Mehrab-Mohseni et al., 2011; Wuestenberg et al., 2014). These findings suggest that BACH1 regulates HCV replication and cytotoxicity of HCV proteins, and may affect drug efficacy.

#### Nonalcoholic steatohepatitis

Nonalcoholic fatty liver disease, which is the leading cause of chronic liver disease, affects the health of more than one billion people. NASH, a severe form of nonalcoholic fatty liver disease, can progress to cirrhosis, HCC, and death (Younossi et al., 2019). A methionine-choline deficient (MCD) diet was fed to wild type (WT) and BACH1(−/−) mice and the WT mice were shown to develop evident hepatic steatosis with a six-fold increase in hepatic triglyceride content; however, BACH1(−/−) mice exhibited negligible hepatic steatosis. Additionally, alanine aminotransferase (ALT) plasma concentrations and hepatic malondialdehyde (MDA) levels increased and peroxisome proliferator-activated receptor α and microsomal triglyceride transfer protein mRNA levels were downregulated in WT mice fed an MCD diet; however, no significant changes were observed for these parameters in BACH1(−/−) mice (Inoue et al., 2011). BACH1 also plays a role in hepatocyte-specific Sirt6-knockout (KO) and WT mice fed a high-fat and high-fructose diet. KO mice presented with increased steatohepatitis and fibrosis via elevated BACH1 expression, because Sirt6 interacts with BACH1, thereby inducing its detachment from the HO-1 promoter (Ka et al., 2017). These studies suggest that BACH1 deficiency plays a hepatoprotective role in NASH.

#### Drug-induced liver injury

Drug-induced liver injury (DILI) is the most common cause of acute liver failure in the United States and the United Kingdom (Lee, 2003; Bernal and Wendon, 2013). DILI is one of the most challenging liver diseases faced by gastroenterologists, as it has a wide variety of clinicopathological phenotypes and lacks specific biomarkers (European Association for the Study of the Liver et al., 2019). Two studies have investigated the role of *Bach1* single nucleotide polymorphisms (SNPs) in anti-tuberculosis drug-induced hepatotoxicity (ATDH) in 870 Chinese and 100 Japanese patients. These studies revealed that *Bach1* SNPs were significantly associated with ATDH; however, this was observed with different tagSNPs (rs372883 and rs1153285 in the Chinese study, and rs2070401 in the Japanese study). The differences in *Bach1* tagSNPs could be attributed to the different tagSNP selection criteria or different allele or genotype frequencies in different patients (Nanashima et al., 2012; Zhang H. et al., 2018). Additionally, *XPO1* encodes the protein Xpo1, which can bind to BACH1 to indirectly influence its antioxidative activity. Patients with the tagSNP rs4430924A in *XPO1* were at a higher risk of developing ATDH than those with rs4430924G (He et al., 2019). SNPs are potential biomarkers for the diagnosis of ATDH.

APAP overdose is a common cause of acute liver injury (Ghanem et al., 2016). Serum HO-1, hepatic BACH1, and NRF2 levels and the nuclear export of NRF2 and BACH1 increase in patients with APAP-induced liver injury. Both vitamin D and omega-3 fatty acids protect against APAP-induced liver injury by decreasing serum HO-1 and hepatic NRF2 and BACH1 levels. Furthermore, omega-3 fatty acids showed preventive effects by promoting the nuclear export and accumulation of BACH1 and NRF2, respectively (Abo El-Magd and Eraky, 2020; Eraky and Abo El-Magd, 2020). These findings indicate that the massive and destructive elevation of BACH1 may contribute to APAP-induced liver injury and decreased BACH1 expression and nuclear translocation may alleviate the extent of liver injury.

#### Sepsis-induced liver injury

Sepsis-induced liver injury is an important independent risk factor for mortality in the intensive care units (Sun J. et al., 2020). Some clinical features of sepsis are attributed to an endotoxin-induced increase in vascular endothelial permeability; therefore, endotoxemia is often used to mimic the hyperinflammation associated with early sepsis and aid the understanding of the molecular mechanisms of sepsis (Aird, 2003; Dickson and Lehmann, 2019). Tanioka et al. (2021) used lipopolysaccharides (LPS) to establish an endotoxin-induced liver-injury rat model. Nuclear BACH1 proteins showed a significant but transient decline by 1 h after LPS injection, followed by a rapid increase to baseline levels by 3 h *Bach1* mRNA elevated shortly after the transient decrease in nuclear BACH1 proteins, then rapidly returned to baseline levels by 24 h. D-galactosamine and LPS were used to establish the endotoxin-induced hepatic injury model in WT and BACH1(−/−) mice. Plasma ALT and aspartate aminotransferase (AST) activities were reduced in BACH1(−/−) mice (Iida et al., 2009). Sepsis was induced by cecal ligation and puncture in WT and BACH1(−/−) mice. In septic mice, knockout BACH1 resulted in increased hepatic HO-1 expression as well as hepatic and pulmonary blood flow, but also attenuated oxidative stress, inflammation, hepatic injury (including liver mitochondrial dysfunction), and mortality. Inhibiting HO-1 activity worsened organ function in KO mice following sepsis (Cai et al., 2022). In summary, BACH1 is a potential therapeutic target in sepsis-induced liver injury.

#### Other types of liver injury

Carbon tetrachloride (CCl_4_) is a toxic agent commonly used to induce acute or chronic liver injury (Unsal et al., 2021). BACH1 expression was upregulated in liver fibrosis and CCl_4_-induced liver injury. San Wei Gan Jiang San, an ancient medicine, alleviates CCl_4_-induced chronic liver injury and fibrosis by reducing BACH1 expression as well as increasing BACH1 nuclear export and NRF2 expression to regulate oxidative stress, including increasing GST, GSH-Px, and HO-1 levels and GSH/GSSG ratio, and reducing MDA levels (Chen et al., 2020). In pulmonary fibrosis, BACH1 expression is upregulated and BACH1 knockdown ameliorates fibrosis and inflammation by inhibiting the ERK signal pathway (Liu Y. et al., 2021). In addition, decreased nuclear BACH1 levels plays a protective role against acute CCl_4_-induced liver injury, hepatitis, and oxidative damage of hepatocytes (Cai et al., 2021). Those suggest that BACH1 contribute to acute and chronic liver injury.

Aflatoxin B1 (AFB1), a mycotoxin that affects humans and animals, is a group 1 human carcinogen; however, the mechanism of toxicity is poorly understood (Marchese et al., 2018). BACH1 deficiency reduces AFB1-induced liver oxidative damage by upregulating antioxidant genes. A kind of small molecule inhibitor of BACH1, named 1-Piperazineethanol, α-[(1,3-benzodioxol-5-yloxy) methyl]-4-(2-methoxyphenyl) (M2), ameliorates AFB1-induced human cell death *in vitro* and liver injury *in vivo* (Zhang et al., 2022). These findings suggest that BACH1 contributes to acute and chronic liver injury.

Arsenic accumulates in organs, particularly in the liver where it may cause liver injury and increase the risk of liver cancer (Palma-Lara et al., 2020). Transcription repressor BACH1 nuclear export increased and the expression of the downstream target genes HO-1, NADPH, and NQO1 increased in inorganic arsenic-induced liver injury samples, whereas BACH1 expression levels showed no change after organic arsenic treatment (Liu D. et al., 2013; Liu D. et al., 2021). Despite these findings, the functions of BACH1 in arsenic-induced liver injury warrants further study.

BACH proteins are associated with aryl hydrocarbon receptor (AhR)-mediated hepatotoxicity, liver ischemia-reperfusion (IR) injury, and radiation induced cytotoxicity. Hepatic BACH1 expression was upregulated in an AhR-mediated hepatotoxic mouse model (Fader et al., 2017). The reduction of BACH1 mediated by miR-27a-5p, increased Bcl-2 and decreased caspase-3 to alleviate apoptosis in liver IR injury (Xing et al., 2018). Microarray analysis showed that the liver is the main organ affected by radiation therapy and BACH2 expression is upregulated during radiotherapy (Karim et al., 2016).

#### Cholestasis

Cholestasis is a disease caused by mechanical obstruction of bile transport or changes in hepatocyte function to produce bile (Salas-Silva et al., 2019). Excess estrogen decreases bile flow, which results in estrogen-induced cholestasis (Zu et al., 2021). Rats given ethinylestradiol for 5 days exhibited cholestasis. HO-1 levels were induced by heme 24 h prior to ethinylestradiol administration. The results showed that heme promoted HO-1 via the NRF2/BACH1 pathway; therefore, cholestasis was reduced in two ways: by stimulating hepatic Mrp3 expression to promote bile flow and increasing urinary bile-acid clearance by up-regulating renal Mrp2/Mrp4 expression or down-regulating renal Mrp3 expression (Muchova et al., 2015). These findings indicate that BACH1 may be a contributing factor for estrogen-induced cholestasis.

#### Primary sclerosing cholangitis

Primary sclerosing cholangitis (PSC) is a relatively rare disease characterized by multifocal biliary strictures with bile duct fibrosis. IBD is present in 70% of patients with PSC and there is a high risk of cholangiocarcinoma and colorectal cancer (CRC) (Dyson et al., 2018). Immunochip analysis of 3,789 PSC cases and 25,079 population controls showed that SNPs of the BACH2 locus, rs56258221, are significantly associated with susceptibility to PSC. This association exhibits a low to moderate linkage disequilibrium (LD) with a BACH2 variant of type 1 diabetes (T1D) and Crohn’s disease (Liu J. Z. et al., 2013). These findings suggest that rs56258221 may serve as a biomarker for PSC and patients with PSC and IBD may be closely associated with the LD of BACH2.

## Intestinal diseases

### Inflammatory bowel disease

IBDs, including ulcerative colitis (UC) and Crohn’s disease (CD), are chronic and progressive disorders associated with genetic, environmental, microbial and immune factors (Agrawal et al., 2021). Bioinformatic analysis predicted BACH1 to be the key transcription factor regulating nine hub genes, which were correlated with Mayo scores in patients with UC (Ding et al., 2021). In addition to decreasing disease activity index, BACH1 depletion dramatically increases HO-1 expression to exert an intestinal mucosal protective effect in a DSS-induced colitis mouse model (Takagi et al., 2018; Dun et al., 2021). Furthermore, BACH1-deficient macrophages exhibit distinctly increased HO-1 levels and manifest the M2 macrophage marker to suppress TNBS-induced colitis (Harusato et al., 2013). BACH1 deficiency in mouse macrophages affects mitochondrial function, including regulation of mitochondrial energy metabolism (increased glycolysis and decreased oxidative phosphorylation), elevated mitochondrial membrane potential and the levels of mitochondrial reactive oxygen species (ROS), and decreased PINK1/Parkin-mediated mitophagy levels. Downregulated expression of BACH1 not only promotes NLRP3 inflammasome activation by regulating inflammatory factor IL-1β expression, but also increases pro-inflammatory cytokines IL-6 and tumor necrosis factor α (TNFα) (Pradhan et al., 2022). Studies indicated that BACH1 may be a candidate gene for target therapy of IBD.

*Bach2* (rs1847472) is a UC susceptibility locus (Romani et al., 2021). Christodoulou et al. identified a potentially deleterious variation of *Bach2* in pediatric patients with UC (Christodoulou et al., 2013). BACH2 is upregulated in UC and significantly increases in signaling by interacting with interleukins. Additionally, target genes of BACH2, including *MMP7, MMP9, AKT3 and GNGT2*, are involved in estrogen signaling. The overexpression of BACH2 probably influences UC by interleukins or estrogen signaling (Ding et al., 2021). Genome-wide meta-analysis demonstrated that rs1847472 of *Bach2* is also a confirmed CD susceptibility locus and is associated with T1D and celiac disease (Franke et al., 2010). Furthermore, rs1847472-C in *Bach2* resulted in an increased risk of nonmelanoma skin cancers (Cushing et al., 2022). A clinical cohort study showed that rs1847472 was also a CD recurrence susceptibility locus after intestinal resection. The rs1847472 may increase the risk of a hyperinflammatory response to gut microbiota after bowel resection (Laffin et al., 2018). Emerging evidence suggests that mucosal immunity plays a central role in intestinal immune homeostasis. IgA production by B cells is a central role in mucosal immunity (Graham and Xavier, 2013). BACH2 promotes immunoglobulin class switch by repressing the regulatory network of plasma-cell gene in B cells (Muto et al., 2010). The function of BACH2 in IBD may be associated with the induction of immunoglobulin class switch. BACH2 forms a transcriptional network with RORγt, FOSL2, AP-1, and RUNX1 to control wound healing. The downregulation of BACH2 regulates the production of CD161+ regulatory T (Treg) cells which enhances wound healing of the colorectal epithelium and is associated with reduced inflammation in IBD (Povoleri et al., 2018). Although BACH2 is closely related to the pathogenesis of UC and CD, more studies are needed to elucidate its roles.

### Celiac disease

Coeliac disease (CeD) is a relatively common and overlooked immune-mediated disorder that occurs in genetically susceptible individuals. The gluten-free diet is currently the only available management plan, but it is not curative or universally effective (Pinto-Sanchez et al., 2021). A study showed that rs10806425 of *Bach2* is strongly implicated in CeD pathogenesis (Medrano et al., 2019). BACH2 expression is significantly reduced in CD4^+^ T cells isolated from the blood of patients with CeD. Differentially expressed genes regulated by BACH2 are highly enriched and over-represented in the above CD4^+^ T cells (Quinn et al., 2015). As a main factor regulating CD4^+^ T cell differentiation, BACH2 controls the efficient formation of Treg cells and inhibits Treg-dependent lethal inflammation. Mild and incompletely penetrating bowel inflammation appeared in some BACH2(−/−) mice (Roychoudhuri et al., 2013). Decreased BACH2 expression has been shown to promote a pro-inflammatory response through lack of efficient Treg cell formation and may contribute to partial CeD and IBD.

### Other intestinal diseases

Non-steroidal anti-inflammatory drugs (NSAIDs) are used worldwide with the most common side effect including gastrointestinal damage called NSAID-associated enteropathy (Scarpignato and Hunt, 2010). In addition to suppressing apoptosis, disruption of BACH1 effectively inhibits inflammatory chemokines such as keratinocyte chemoattractant (KC), MIP1α, and MCP1 to ameliorate indomethacin-induced intestinal injury in mice (Harusato et al., 2009; Harusato et al., 2011).

Intestinal ischemia-reperfusion (IR) injury is a complex disease with high morbidity and mortality (Ding et al., 2020). The downregulation of BACH1 expression distinctly reduces the levels of myeloperoxidase, hemoglobin, KC, luminal protein, and TNFα in intestinal IR injury mice. Simultaneously, this decreases PMN infiltration into the small intestine and attenuates the activation of NF-κB to reduce the levels of adhesion proteins, E-selectin and ICAM-1. Attenuation of myeloperoxidase and luminal protein expression can be reversed by reducing HO-1 expression (Katada et al., 2022). A similar mechanism involving BACH1 has been observed in cerebral IR injury (Yu et al., 2020). These findings reveal that BACH1 deficiency alleviates intestinal IR injury by increasing HO-1 expression. BACH2 deletion facilitates intestinal epithelial regeneration by promoting DNA repair of intestinal crypt cells in radiation-induced intestinal injury (Li et al., 2021).

## Pancreatic diseases

### Chronic pancreatitis

Chronic pancreatitis (CP) is a multifactorial fibroinflammatory syndrome of the exocrine pancreas. CP incidence has been increasing gradually worldwide, with no effective curative therapy (Beyer et al., 2020). BACH2 expression downregulates in the pancreas tissue and circulation of patients with CP. The repression of BACH2 expression leads to the polarization of T-helper cells toward Th17 cells and increased inflammation in patients with CP. Additionally, the rs9111-TT genotype in the 5′-UTR region of BACH2 decreases the expression of BACH2 and is associated with clinical features of advanced CP (Sasikala et al., 2018). This suggests that BACH2 plays an important role in protecting from CP by regulating immunity.

### Conclusion of benign diseases of the digestive system

**FIGURE 4:**
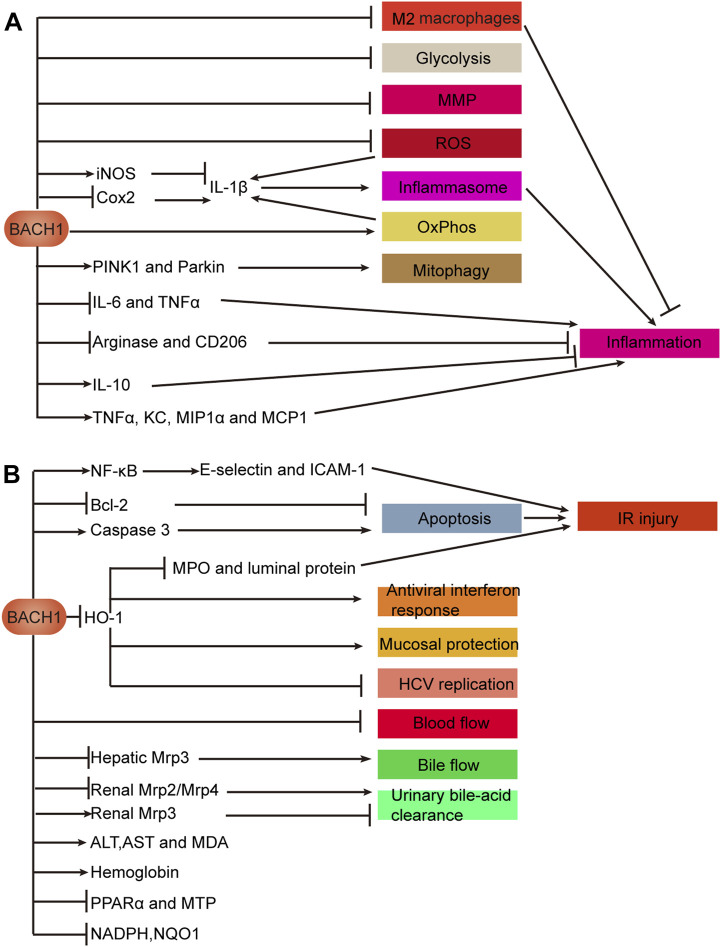
Mechanisms of BACH1 in benign diseases of the digestive system. **(A)** BACH1 regulates inflammation. **(B)** Effects of BACH1 on biological processes such as oxidative stress. A In macrophages, BACH1 inhibits glycolysis, MMP, and ROS and induces mitophagy via the PINK1/Parkin pathway. BACH1 decreases the number of M2 macrophages to promote inflammation. Inflammatory factor IL-1β is regulated by BACH1 in a variety of ways, as illustrated. IL-1β promotes the activation of the NLRP3 inflammasome to promote inflammation. Furthermore, BACH1 regulates inflammation by promoting or inhibiting anti-inflammatory markers, including CD206, arginase, and IL-10 and pro-inflammatory cytokines, including IL-6, TNF-α, KC, MIP1α, and MCP1. B BACH1 promotes IR injury involving the NF-κB pathway, apoptosis, and HO-1-mediated protection. BACH1 plays a role in viral replication, anti-interferon response, mucosal protection, and IR injury through transcriptional repression of HO-1. In addition to inhibiting urinary bile-acid clearance and bile and blood flow, BACH1 affects the levels of some proteins and metabolites that reflect liver function. Abbreviations: keratinocyte chemoattractant (KC); ischemia and reperfusion (IR); mitochondrial membrane potential (MMP); reactive oxygen species (ROS); oxidative phosphorylation (OxPhos); microsomal triglyceride transfer protein (MTP); peroxisome proliferator-activated receptor α (PPARα); ischemia-reperfusion (IR).

BACH proteins play an indispensable role in inhibition of HCV replication, intestinal mucosal protection, inflammation regulation, alleviation of steatohepatitis and fibrosis, and digestive-system damage caused by drugs, poisons, or ischemia-reperfusion. BACH proteins affect benign diseases mainly through oxidative stress and inflammation (Figures 4A, B).

In HCV, DILI, cholestasis, IBD, IR injury and other diseases, BACH1 transcriptionally inhibits HO-1 to mediate oxidative stress response, which causes aggravated organ function damage and a rise in related indicators. In addition to affecting multiple diseases through similar mechanisms, BACH1 affects the same disease through multiple mechanisms. IR injury is regulated by three BACH1-mediated pathways: HO-1 transcriptional repression, NF-Κb activation, and promotion of apoptosis.

BACH1 regulates the biological function of macrophages and affects the expression of anti-inflammatory and pro-inflammatory factors, thereby inhibiting or promoting inflammation in IBD and other inflammation-related diseases. Interestingly, BACH1 deficiency promotes pro-inflammatory cytokine TNFα expression in bone marrow macrophages but suppresses TNFα expression in intestinal macrophages. The regulation of TNFα by BACH1 may be affected by the microenvironment in which macrophages are located. BACH2 regulates inflammation and the differentiation of T cells which affect inflammation or wound healing in IBD, CeD and CP.

### BACH proteins and cancers of the digestive system

By 2040, the global cancer burden is expected to reach 28.4 million cases (Sung et al., 2021). BACH transcription factors, especially BACH1, are involved in the proliferation, metastasis, angiogenesis, metabolism, and chemotherapy resistance of many cancers (Padilla and Lee, 2021). In this section, we focus on discussing the roles of BACH proteins in digestive-system cancers.

### Esophageal cancer

Esophageal cancer ranks seventh as the most prevalent type of cancer and sixth in mortality worldwide. Esophageal squamous cell carcinoma (ESCC) is a common histological subtype of esophageal cancer (Sung et al., 2021). BACH1 is involved in LncRNA SNHG8/miR-1270/BACH1 axis in esophageal cancer. LncRNA SNHG8 decreases the level of miR-1270 to increase the expression of BACH1, thereby promoting cancer progression (Wu et al., 2022). BACH1 expression is increased in ESCC tissues compared with that of healthy esophageal epithelial tissues. BACH1 induces epithelial-mesenchymal transition (EMT) by activating CDH2, SNAI2, and vimentin, and facilitates angiogenesis by upregulating the transcriptional activity of vascular endothelial growth factor C, thereby promoting the proliferation and metastasis of ESCC cells (Zhao et al., 2021). BACH1 inhibits Wnt/β-catenin pathway by recruiting HDAC1 to affect transcription of TCF4-targeted genes and impairing the interaction between β-catenin and p300/CBP or TCF4. In this way, BACH1 suppresses angiogenesis after ischemic injury (Jiang et al., 2015). BACH1 also inhibits angiogenesis in pancreatic cancer (Huang et al., 2018). It is suggested that the effect of BACH1 on angiogenesis is different in different diseases. BACH1 inhibits the biosynthesis of monounsaturated fatty acids (MUFAs), especially oleic acid, by transcriptionally inhibiting the *SCD1* gene in ESCC cells. In this way, BACH1 induces ferroptosis. Thus, BACH1 suppresses hematogenous metastasis *in vivo* due to high levels of iron ions and oxidative stress in the blood. BACH1 promotes lymph node metastasis *in vivo* because lymph is rich in MUFAs such as oleic acid. The concentration gradient of MUFA between the primary lesion and the lymph resulted in the chemoattraction of ESCC cells to induce metastasis. In addition, MUFAs act as a protective coating around ESCC cells, protecting them from ferroptosis (Xie et al., 2023). Further mechanistic studies are required to elucidate the function of BACH1 in esophageal cancer.

#### Gastric cancer

Gastric cancer (GC) is the common malignant tumor and cause of cancer-related death (Smyth et al., 2020). Fang et al. showed using bioinformatics analyses that BACH1 overexpression indicated good prognosis in patients with GC (Fang and Lu, 2020). A recent study revealed that BACH1 facilitates the polarization of macrophages towards the M2 phenotype by activating Wnt1 and promotes macrophage-dependent GC progression (Yang et al., 2022). These somewhat contradictory results suggest that the role of BACH1 in GC is complex and may be related to the tumor immune microenvironment.

Bioinformatics analyses showed that BACH2 increased and was positively correlated with short survival time in patients with GC of microsatellite instability-high status (Tian et al., 2020). Methylation of the BACH2 promoter was reported to be increased in approximately half of patients with GC and associated with reduced expression of BACH2. Downregulated expression of BACH2 occurred in significantly less frequencies in the diffuse-type gastric cancers compared with that of the intestinal-type gastric cancers. Moreover, BACH2 deficiency has been shown to promote GC cell proliferation *in vitro* (Haam et al., 2014). These findings indicate that BACH2 is expressed at different levels and may play a positive or negative role in different GC subtypes. Therefore, BACH proteins are worthy of further study to reveal their role in gastric tumorigenesis.

#### Colorectal cancer

CRC is the second most common cause of cancer-related death, after lung cancer, with more than 1.9 million cases and 930,000 deaths annually worldwide. CRC accounts for approximately one in ten cancer cases and deaths globally. The primary cancer site of CRC is mainly the colon (Sung et al., 2021). A study showed that *Bach1* mRNA levels were more upregulated in CRC than in paracancerous tissues (Chang et al., 2016; Chang et al., 2020). Immunohistochemical results showed that BACH1 expression was decreased in CRC tissues compared with that of adjacent healthy tissues, whereas no significant difference was observed in the expression of BACH1 in CRC tissues compared with that of distant healthy tissues (Chang et al., 2013). Another study demonstrated that BACH1 expression was lowest in adenomas, high in colon cancer tissues, and highest in normal tissues adjacent to colon cancer tissues. Increased BACH1 levels were positively associated with tumor progression in colon cancer (El-Deek et al., 2019). BACH1 expression is dynamically altered and likely to be epigenetically modified in colorectal malignant transformation and tumor progression.

In colon cancer, BACH1 expression increases and promotes cancer cell migration by increasing the expression of metastasis-related genes including MMP-1, MMP-9, MMP-13, SNAIL1, CXCR4, and HMGA2. The increased metastasis-related genes may be due to the BACH1-mediated decrease in miR-34a and let-7a (Davudian et al., 2016). The overexpression of BACH1 enhances CRC cell proliferation, migration, and invasion and may be associated with upregulated levels of CD31, vimentin, and CRC4 (Zhu et al., 2018). BACH1 knockdown inhibits STARD8 and TIAM2 expression at the transcriptional level, thereby suppressing CRC metastasis (Chen et al., 2022). Long non-coding RNA NEAT1 directly binds to let-7g-5p, which targets and upregulates BACH1 to facilitate EMT and cell growth of colon cancer (Gao et al., 2021). However, one study showed that the proliferative capacity of colon cancer cells was not affected by BACH1 knockdown *in vitro* (Davudian et al., 2016). More studies are needed to clarify the role of BACH1 in CRC proliferation. The association between BACH1 expression and mitochondrial function is different in CRC with or without metastasis (Chang et al., 2020). BACH1 negatively regulates mitochondrial metabolism in cancer cells by affecting transcription of mitochondrial respiratory chain genes and glucose utilization in the citric acid cycle (Lee et al., 2019). These findings indicate that BACH1 promotes metastasis by potentially affecting mitochondrial function. In addition, circBACH1 augmented the proliferation, migration, and metastasis of CRC and suppressed apoptosis by inhibiting et-7a-5p, which targets CREB5 and increased CREB5 expression (Li et al., 2020).

Gastrin releasing peptide (GRP) and its receptor (GRPR) are morphogenetic factors, which keep tumor cells at high degree of differentiation and reduce tumor metastasis ability. BACH2 expression is specifically downregulated when GRP/GRPR are aberrantly upregulated in colon cancer (Ruginis et al., 2006). BACH2 proteins promote immune homeostasis and long-term tumor immunosuppression by driving the quiescence and durable maintenance of resting Treg cells. In this way, BACH2 promotes tumor growth in colorectal adenocarcinoma (Grant et al., 2020). In addition to affecting adaptive immunity, deficiency of BACH2 expression promotes natural killer (NK) cell maturation, regulates its function, and suppresses tumor metastasis in mice (Li et al., 2022). In summary, both BACH1 and BACH2 play promoting roles in the progression of CRC.

#### Liver cancer

The global incidence of liver cancer is projected to exceed 1 million cases by 2025. Hepatocellular carcinoma (HCC) accounts for approximately 80% of the total liver-cancer cases (Forner et al., 2018). The expression of BACH1 was increased in HCC tissues and positively correlated with worse prognosis. Increased BACH1 expression facilitated the proliferation and metastasis of HCC by directly targeting IGF1R and PTK2. Furthermore, IGF2, which can interact with IGF1R, promotes BACH1 expression upregulation via the ERK1/2/ETS1 signaling pathway to form a positive feedback loop in HCC progression (Xie et al., 2022). BACH1 also facilitated transcription of HK2 which promotes glycolysis-induced HCC metastasis (Zhao et al., 2022). A recent study showed that BACH1 promoted cellular inflammatory factors and suppressed autophagy in HCC by negatively regulating the p53 pathway (Sun et al., 2021). In contrast to BACH1, upregulated BACH2 expression may enhance apoptosis in NASP-depleted HCC cells (Kang et al., 2018).

BACH1 is an essential component of Ten-eleven translocation 1 (TET1)/miR-34a/BACH1 axis, through which TET1 negatively modulates BACH1 by demethylating and activating miR-34a (Sun et al., 2021). BACH1 proteins facilitate HCC proliferation, migration, invasion, EMT progress, and cell cycle. LncRNA TRG-AS and Lnc712 interact with miR-4500 and miR-142-3p, respectively, to induce an increase in BACH1 expression (Sun X. et al., 2020; Cui and Ni, 2020). Paradoxically, BACH1 regulated by miR-25-3p and let-7a-5p induces the accumulation of ROS while suppressing cell proliferation (Wang et al., 2020). BACH1 binds to the two identified binding motifs (13271nt and 19595nt) in TKT to inhibiting TKT expression. The knockdown of TKT expression affects HCC cells in multiple ways; it sensitizes HCC cells to sorafenib, suppresses cell proliferation and lung metastasis, decreases glucose uptake *in vitro*, alters glutathione metabolism, increases intracellular ROS levels, and induces oxidative stress-related G1 phase arrest (Xu et al., 2016).

One study reported that circBACH1 accelerated cell cycle progression to promote HCC growth by interacting with HuR to block the translation of p27, which regulates cell cycle (Liu et al., 2020). CircBACH1 was also reported to promote HBV replication and HCC development by sponging miR-200a-3p to downregulate MAP3K2 expression (Du et al., 2022). Collectively, circBACH1 is an oncogenic factor of HCC and BACH1 plays an important role in HCC progression, metabolism, oxidative stress, and chemoresistance.

#### Cholangiocarcinoma (CCA)

CCA is a highly aggressive and chemoresistant malignant tumor of the digestive system (Rodrigues et al., 2021). A recent bioinformatics analysis showed that BACH1 expression decreased in CCA (Liu Z. et al., 2022). Increased BACH1 expression, mediated by the nuclear accumulation of FOXO1, represses the transcription of genes encoding proteasome subunits. Decreased PTEN expression downregulates the nuclear translocation of FOXO1 via the PI3K/AKT pathway, resulting in dependency on the proteasome for CCA cell growth and survival and sensitivity to proteasome inhibitors. This mechanism may be widespread in many cancers (Jiang T.-Y. et al., 2020). This study suggests that BACH1 is a potential biomarker and therapeutic target for resistance to proteasome inhibitor chemotherapy in CCA and other cancers.

#### Pancreatic cancer

Pancreatic cancer is a clinically challenging cancer. The prognosis of pancreatic cancer is very poor, with a median survival of less than a year after treatment (Wood et al., 2022). Therefore, there is an urgent need for efficient treatment methods, such as molecular targeted therapy.

BACH1 is mainly found in islet cells of the healthy human pancreas, acinar and ductal cells of CP, and ductal and stromal cells of pancreatic ductal adenocarcinoma (PDAC). Additionally, BACH1 is present in urine exosomes. BACH1 is dynamically expressed in cancer development from the early lesions to invasive stages and are more highly expressed in cancer tissues than in normal tissues. Moreover, BACH1 levels are inversely correlated with prognosis in patients with PDAC (Kim et al., 2021). BACH1 increases labile iron by inhibiting the expression of the ferritin subunit genes *FTH1* and *FTL*. On the one hand, labile iron promotes EMT by inhibiting the expression of the epithelial gene *E-cadherin.* On the other hand, labile iron promotes BACH1 degradation by promoting Fbox22 expression. BACH1 and labile iron form negative feedback regulation (Liu et al., 2022). BACH1 has been shown to promote cancer metastasis by promoting EMT. BACH1 promotes EMT by suppressing the expression of E-cadherin, FOXA1, and tight junction proteins (OCLN, CLDN3, CLDN4) and promoting the expression of vimentin and SNAI2. ChIP-sequencing analysis showed that BACH1 directly inhibited the expression of CLDN3, CLDN4, FOXA1, and PKP2, whereas it directly activated SNAI2 expression (Sato et al., 2020). The expression of E-cadherin is induced by FOXA1 and inhibited by SNAI2 (Song et al., 2010; Carpenter et al., 2015). *In vitro* and *in vivo* experiments showed that BACH1 deficiency had no effect on proliferation (Sato et al., 2020). Nevertheless, one study showed that BACH1 expression decreased in patients with PDAC and BACH1 deficiency facilitated the growth of PDAC cells and angiogenesis, most likely by upregulating HO-1 to increase the expression of eNOS, VEGF, and HIF1A, evoke oncogenic AKT and ERK signaling, and inhibit PTEN expression. BACH1 promotes the expression of epithelial cell markers E-cadherin and ZO-1, and inhibits the expression of mesenchymal cell markers ZEB1, vimentin, and Slug, as well as stemness markers ABCG2 and ALDH1. This study also demonstrated that the rs372883C allele in the 3′-untranslated region (3′-UTR) of *Bach1* resulted in evidently higher BACH1 levels than the rs372883T allele in both cancer and normal tissues. Additionally, patients with PDAC carrying the rs372883T allele were more resistant to gemcitabine and had worse overall survival compared with those carrying the rs372883C allele. It is suggested that patients with BACH1 deficiency were resistant to gemcitabine and had shorter survival times (Huang et al., 2018). Interestingly, BACH1 deficiency promotes E-cadherin expression in AsPC−1 (derived from ascitic fluid of pancreatic cancer) and SW1990 cells (derived from splenic metastases of pancreatic cancer), but inhibits E-cadherin expression in CFPAC-1 (derived from liver metastases of pancreatic cancer) and BXPC-3 cells (derived from orthotopic pancreatic cancer) (Huang et al., 2018; Sato et al., 2020). It indicated that BACH1 may have different effects on EMT-related genes in pancreatic cancer cell lines of different origins. Further mechanistic studies are needed to elucidate the function of BACH1 in pancreatic cancer.

### Conclusion of cancers of the digestive system

**FIGURE 5:**
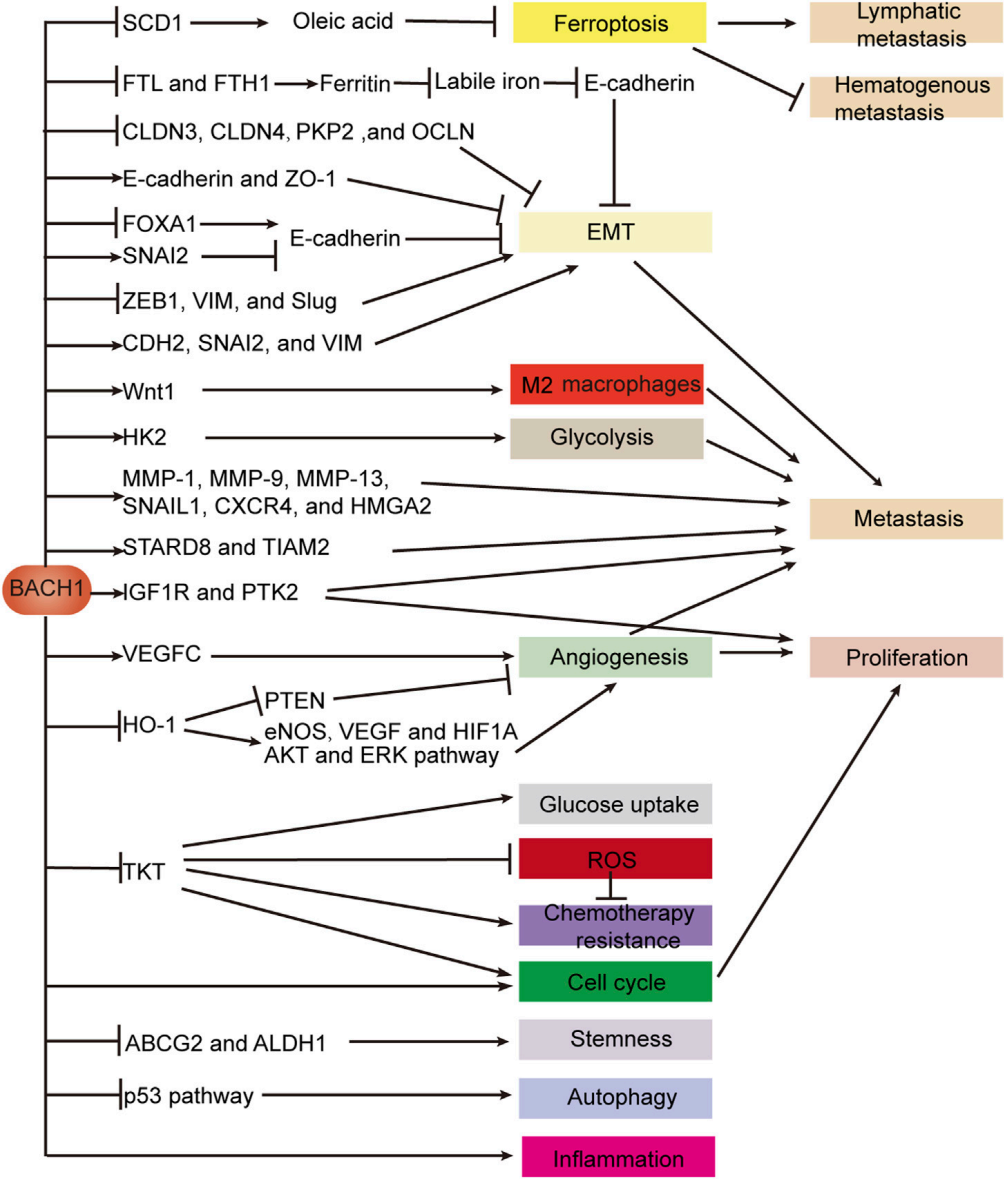
BACH1 regulates gene expression in digestive system cancers. BACH1 regulates EMT by activating or silencing genes which are critical for epithelial or mesenchymal cell structure. BACH1 also targets genes encoding ferritin subunits and indirectly promotes EMT by increasing labile iron. BACH1 induces ferroptosis to drive lymphatic metastasis and inhibit hematogenous metastasis by directly suppressing the transcription of SCD1, which is essential for oleic acid production. The expression of metastasis-related genes is indirectly promoted or transcriptionally activated by BACH1. BACH1 not only promotes tumor angiogenesis through VEGFC, but also transcriptionally inhibits HO-1 to inhibit tumor angiogenesis. BACH1 regulates multiple biological steps by directly targeting genes such as TKT and HK2 or indirectly regulating pathways such as the p53 pathway. Abbreviations: vimentin (VIM); epithelial-mesenchymal transition (EMT).

BACH proteins regulate ferroptosis, EMT, angiogenesis, stemness, autophagy, chemotherapy resistance, metabolism, the immune microenvironment, proliferation, and metastasis by binding to the promoter of the target gene to affect gene expression or indirectly affecting gene expression (Figure 5). We focus on the mechanism of BACH1 in EMT, angiogenesis, proliferation, and metastasis. BACH1 regulates EMT by controlling the expression of EMT key genes such as CLDN3 or inhibiting the expression of ferritin subunit genes to decrease E-cadherin expression. The effect of BACH1 on EMT-related genes may be quite different in cell lines of different origins. For example, BACH1 inhibits EMT by decreasing vimentin expression in pancreatic cancer but promotes EMT by increasing vimentin expression in esophageal cancer. Angiogenesis is inhibited by the BACH1/HO-1 axis and promoted by the BACH1/VEGFC pathway. BACH1 regulates cancer proliferation by targeting IGF1R and PTK2 and affecting angiogenesis and cell cycle. Cell cycle is promoted by BACH1 and TKT which is transcriptionally suppressed by BACH1. BACH1 promotes metastasis by inducing metastasis-related gene expression, HK2-mediated glycolysis, Wnt1-mediated M2 macrophage polarization, EMT, angiogenesis, and ferroptosis. BACH2 maintains immune homeostasis and durable tumor immunosuppression and induces cancer progression by regulating Treg and NK cells in gastrointestinal tumors.

## Conclusion

BACH transcription factors are important regulators of pathophysiology in the digestive system. The regulatory role of BACH proteins is dual in a variety of processes such as inflammation, EMT, angiogenesis, cell cycle, and macrophage polarization. For example, BACH1 knockdown promotes M2 macrophage polarization to alleviate colitis and inhibits M2 macrophage polarization to inhibit GC metastasis. ROS production is promoted by BACH1 in cancer cells but inhibited by BACH1 in macrophages. Different cellular functions regulated by BACH proteins are interconnected. For example, BACH1-induced ferrroptosis promotes lymphatic metastasis and inhibits hematogenous metastasis. In inflammation, EMT, and other conditions, BACH proteins regulate the same molecule differently in different cell tissues. For example, BACH1 increases the expression of hepatic Mrp3 and decreases the expression of renal Mrp3. The BACH1/HO-1 pathway plays a role in both benign and malignant diseases of the digestive system. In addition to inhibiting HCV replication and IR injury and promoting mucosal protection and antiviral response, BACH1-mediated HO-1 upregulates the expression of VEGF and other molecules and downregulates PTNE expression to regulate tumor angiogenesis. Although BACH2 has been reported to affect CP, IBD, and cancer by regulating adaptive immunity, the specific functions of BACH2 are unclear.

BACH protein expression levels appear to be related to the severity of IBD, CeD, CP, and the prognosis of patients with cancer. Therefore, these proteins may serve as biomarkers and therapeutic targets for digestive diseases, such as IBD, and tumor progression and prognosis. In addition to BACH expression levels, SNPs in *Bach* genes are susceptibility loci for IBD, CeD, PSC, ADTH, CP, and PDAC and may serve as biomarkers at the genetic level. Currently, *Bach2* SNPs are associated with autoimmune diseases. However, the causal genetic variants within each susceptibility locus are unclear. Mouri et al. demonstrated an efficient method to preferentially select variants and study their relevant roles (Mouri et al., 2022). This will be useful to better characterize the effect of these SNPs on disease.

**FIGURE 6:**
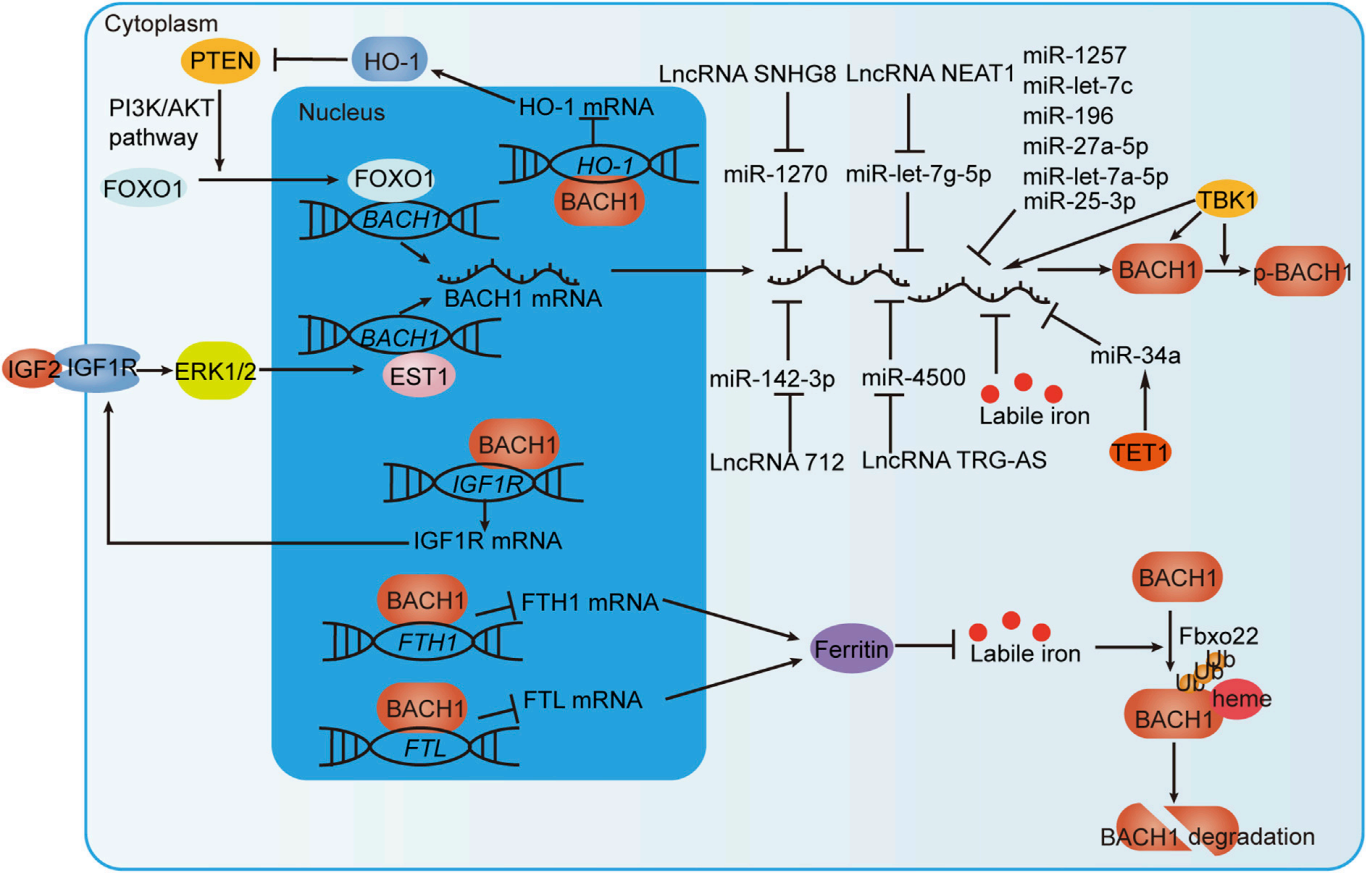
BACH1 regulation by transcriptional, post-transcriptional and post-translational mechanisms. IGF1R interacts with IGF2 to promote the transcription of BACH1 via the ERK1/2/EST1 pathway. In addition, BACH1 targets the *IGF1R* gene to form a positive feedback loop that continuously promotes BACH1 expression. BACH1 increases PNET expression by suppressing HO-1 expression at the transcriptional level. PNET induces BACH1 transcription through transcription factor FOXO1. BACH1 and PNET also form positive feedback. BACH1 mRNA can be directly negatively regulated by miRNAs but also indirectly regulated by TET1 protein or LncRNAs. TBK1 phosphorylates BACH1 and increases BACH1 mRNA and protein levels. Labile iron inhibits the level of BACH1 mRNA and promotes FBXO22-dependent BACH1 degradation. Moreover, BACH1, which targets the FTH1 and FTL genes encoding ferritin subunits, reduces ferritin expression to increase the level of labile iron. In this way, BACH1 and iron form negative feedback.

Pharmacological studies have identified some regulators of BACH. For example, hemin specifically degrades BACH1 with negligible toxicity, inhibits breast cancer growth, and is used to treat patients with acute porphyria (Sun et al., 2002; Chiabrando et al., 2014; Bissell et al., 2017; Lee et al., 2019; Lignitto et al., 2019). Table 2 summarizes some novel molecular compounds capable of modifying the expression and activities of BACH, as well as their function in related diseases. *Bach1* gene is transcriptionally repressed by FOXO1 and EST1. BACH1 mRNA and protein levels are affected by miRNAs, labile iron, LncRNAs, TBK1 protein, and TET1 protein. BACH1 protein is also subject to post-translational regulation by TBK1 and labile iron. In addition, BACH1 forms positive feedback with PTEN or IGF1R and negative feedback with labile iron (Figure 6). These findings provide new ideas for the development of agonists or antagonists targeting BACH in the digestive system.

**TABLE 2:** BACH1 regulator and their effect in the diseases.

Regulator	Disease	Pharmacological function	References
Degrader Hemin	Breast cancer	Promotes the degradation of BACH1	Lee et al. (2019); Lu et al. (2021)
		Alters metabolic pathways which are downstream of BACH1 Increases sensitivity of cancer cells against mitochondrial inhibitors including metformin and AVO	
		Inhibits breast cancer growth *in vivo and vitro*	
	Lung cancer	Promotes the degradation of BACH1	Lignitto et al. (2019)
		Suppresses lung cancer metastasis *in vivo and vitro*	
Degrader	Breast and lung cancer	Promotes the degradation of BACH1 in breast and lung cancer	Moreno et al. (2022)
TBE56 (50-fold more potent than hemin)		Suppresses breast cancer cell metastasisin *in vitro*	
Inhibitor	Aflatoxin B1 (AFB1)-induced liver injury	Inhibits BACH1	Marchese et al. (2018)
M2		Suppresses cell death *in vitro*	
		Improves symptoms of weight loss and liver injury *in vivo*	
Inhibitor HPP (HPP-A, HPP-B, HPP-C, HPP-E and HPP-4382)	Bone destructive diseases such as rheumatoid arthritis	Suppresses BACH1 activity	Attucks et al. (2014); Wada et al. (2020)
		Hinders binding to the HMOX1 E2 enhancer *in vitro*	
		Inhibits RANKL-mediated osteoclastogenesis and lipopolysaccharide-induced bone destruction	
Inhibitor benzimidazole	Parkinson	Inhibits BACH1	Ahuja et al. (2021)
		Plays a neuroprotective role in mouse models	
Inhibitor	Huntington’s disease	Inhibits BACH1	Casares et al. (2020)
Isomeric		Activates NRF2	
O-methylcannabidiolq-uinones		Plays a neuroprotective role *in vitro*	
Inhibitor	Lung cancer	Activates NRF2	Casares et al. (2022)
CDDO-Me and CDDO-TFEA)		Reduces BACH1 nuclear levels while accumulating its cytoplasmic form	
		Impairs lung cancer cell invasion *in vitro*	
Agonists myricetin	Cardiac hypertrophy	Upregulates BACH2 expression	Jiang et al. (2022)
		Downregulates the mRNA levels of hypertrophic marker*Bnp and Myh7*	
		Has a BACH2-dependent protective effect *in vivo* and *in vitro*	

Abbreviations: oleanane triterpenoid 2-cyano-3, 12-dioxooleana-1, 9 (11)-dien-28-oic acid (CDDO); 1-Piperazineethanol, α-[(1,3-benzodioxol-5-yloxy) methyl] -4-(2-methoxyphenyl) (M2); High point pharmaceuticals (HPP).

There has been some progress in understanding BACH proteins. However, many important questions remain. For example, both BACH1 and BACH2 are expressed in the same tissues such as gut, but their temporal and spatial relationships have not been clearly defined. In digestive diseases and tumors, further studies are required to elucidate the mechanisms of action of BACH proteins and aid in the search for valuable regulators targeting BACH proteins to treat diseases.
